# Supplementary material for: HIVprotI: an integrated web based platform for prediction and design of HIV proteins inhibitors
Source: J Cheminform. 2018 Mar 9;10:12. doi: 10.1186/s13321-018-0266-y (PMC5845081; doi:10.1186/s13321-018-0266-y)
Supplement: Supplementary file 2 — Additional file 2. Source code of HIVProtI web server. [file 13321_2018_266_MOESM2_ESM.zip › HIVprotI_Source-code/hivprotI/weka-3-6-6/doc/weka/core/AlgVector.html]

AlgVector


---


|  |  |  |
| --- | --- | --- |
| **Overview** \| **Package** \| **Class** \| **Tree** \| **Deprecated** \| **Index** \| **Help** \| **Weka's home** | |  |
| **PREV CLASS**   **NEXT CLASS** | **FRAMES**    **NO FRAMES**     **All Classes** |  |
| SUMMARY: NESTED \| FIELD \| CONSTR \| METHOD | DETAIL: FIELD \| CONSTR \| METHOD |  |


---


## weka.core Class AlgVector

```
java.lang.Object
  weka.core.AlgVector
```

**All Implemented Interfaces:**: java.io.Serializable, java.lang.Cloneable, RevisionHandler

---

``` public class AlgVector extends java.lang.Object implements java.lang.Cloneable, java.io.Serializable, RevisionHandler ```

Class for performing operations on an algebraic vector
of floating-point values.

**Version:**
:   $Revision: 1.10 $

**Author:**
:   Gabi Schmidberger (gabi@cs.waikato.ac.nz)

**See Also:**: Serialized Form

---

**Constructor Summary**

`AlgVector(double[] array)`
  
          Constructs a vector using a given array.

`AlgVector(Instance instance)`
  
          Constructs a vector using an instance.

`AlgVector(Instances format,
java.util.Random random)`
  
          Constructs a vector using a given data format.

`AlgVector(int n)`
  
          Constructs a vector and initializes it with default values.


| **Method Summary** | |
| --- | --- |
| `AlgVector` | `add(AlgVector other)`             Returns the sum of this vector with another. |
| `void` | `changeLength(double len)`             Changes the length of a vector. |
| `java.lang.Object` | `clone()`             Creates and returns a clone of this object. |
| `double` | `dotMultiply(AlgVector b)`             Returns the inner (or dot) product of two vectors |
| `Instance` | `getAsInstance(Instances model, java.util.Random random)`             Gets the elements of the vector as an instance. |
| `double` | `getElement(int index)`             Returns the value of a cell in the matrix. |
| `double[]` | `getElements()`             Gets the elements of the vector and returns them as double array. |
| `java.lang.String` | `getRevision()`             Returns the revision string. |
| `static void` | `main(java.lang.String[] args)`             Main method for testing this class, can take an ARFF file as first argument. |
| `double` | `norm()`             Returns the norm of the vector |
| `void` | `normVector()`             Norms this vector to length 1.0 |
| `int` | `numElements()`             Returns the number of elements in the vector. |
| `void` | `scalarMultiply(double s)`             Computes the scalar product of this vector with a scalar |
| `void` | `setElement(int index, double value)`             Sets an element of the matrix to the given value. |
| `void` | `setElements(double[] elements)`             Sets the elements of the vector to values of the given array. |
| `AlgVector` | `substract(AlgVector other)`             Returns the difference of this vector minus another. |
| `java.lang.String` | `toString()`             Converts a vector to a string |

**Methods inherited from class java.lang.Object**

`equals, getClass, hashCode, notify, notifyAll, wait, wait, wait`

**Constructor Detail**

### AlgVector

```
public AlgVector(int n)
```

:   Constructs a vector and initializes it with default values.

    **Parameters:**: `n` - the number of elements

---


### AlgVector

```
public AlgVector(double[] array)
```

:   Constructs a vector using a given array.

    **Parameters:**: `array` - the values of the matrix

---


### AlgVector

```
public AlgVector(Instances format,
                 java.util.Random random)
          throws java.lang.Exception
```

:   Constructs a vector using a given data format.
    The vector has an element for each numerical attribute.
    The other attributes (nominal, string) are ignored.
    Random is used to initialize the attributes.

    **Parameters:**: `format` - the data format to use: `random` - for initializing the attributes **Throws:**: `java.lang.Exception` - if something goes wrong

---


### AlgVector

```
public AlgVector(Instance instance)
          throws java.lang.Exception
```

:   Constructs a vector using an instance.
    The vector has an element for each numerical attribute.
    The other attributes (nominal, string) are ignored.

    **Parameters:**: `instance` - with numeric attributes, that AlgVector gets build from **Throws:**: `java.lang.Exception` - if instance doesn't have access to the data format or no numeric attributes in the data


**Method Detail**

### clone

```
public java.lang.Object clone()
                       throws java.lang.CloneNotSupportedException
```

:   Creates and returns a clone of this object.

    :   **Overrides:**: `clone` in class `java.lang.Object`
    :   **Returns:**: a clone of this instance. **Throws:**: `java.lang.CloneNotSupportedException` - if an error occurs

---


### getElement

```
public final double getElement(int index)
```

:   Returns the value of a cell in the matrix.

    :   **Parameters:**: `index` - the row's index **Returns:**: the value of the cell of the vector

---


### numElements

```
public final int numElements()
```

:   Returns the number of elements in the vector.

    :   **Returns:**: the number of rows

---


### setElement

```
public final void setElement(int index,
                             double value)
```

:   Sets an element of the matrix to the given value.

    :   **Parameters:**: `index` - the elements index: `value` - the new value

---


### setElements

```
public final void setElements(double[] elements)
```

:   Sets the elements of the vector to values of the given array.
    Performs a deep copy.

    :   **Parameters:**: `elements` - an array of doubles

---


### getElements

```
public double[] getElements()
```

:   Gets the elements of the vector and returns them as double array.

    :   **Returns:**: an array of doubles

---


### getAsInstance

```
public Instance getAsInstance(Instances model,
                              java.util.Random random)
                       throws java.lang.Exception
```

:   Gets the elements of the vector as an instance.
    !! NON-numeric data is ignored sofar

    :   **Parameters:**: `model` - the dataset structure to fit the data to: `random` - in case of nominal values a random label is taken **Returns:**: an array of doubles **Throws:**: `java.lang.Exception` - if length of vector is not number of numerical attributes

---


### add

```
public final AlgVector add(AlgVector other)
```

:   Returns the sum of this vector with another.

    :   **Parameters:**: `other` - the vector to add **Returns:**: a vector containing the sum.

---


### substract

```
public final AlgVector substract(AlgVector other)
```

:   Returns the difference of this vector minus another.

    :   **Parameters:**: `other` - the vector to subtract **Returns:**: a vector containing the difference vector.

---


### dotMultiply

```
public final double dotMultiply(AlgVector b)
```

:   Returns the inner (or dot) product of two vectors

    :   **Parameters:**: `b` - the multiplication matrix **Returns:**: the double representing the dot product

---


### scalarMultiply

```
public final void scalarMultiply(double s)
```

:   Computes the scalar product of this vector with a scalar

    :   **Parameters:**: `s` - the scalar

---


### changeLength

```
public void changeLength(double len)
```

:   Changes the length of a vector.

    :   **Parameters:**: `len` - the new length of the vector

---


### norm

```
public double norm()
```

:   Returns the norm of the vector

    :   **Returns:**: the norm of the vector

---


### normVector

```
public final void normVector()
```

:   Norms this vector to length 1.0

---


### toString

```
public java.lang.String toString()
```

:   Converts a vector to a string

    :   **Overrides:**: `toString` in class `java.lang.Object`
    :   **Returns:**: the converted string

---


### getRevision

```
public java.lang.String getRevision()
```

:   Returns the revision string.

    :   **Specified by:**: `getRevision` in interface `RevisionHandler`
    :   **Returns:**: the revision

---


### main

```
public static void main(java.lang.String[] args)
                 throws java.lang.Exception
```

:   Main method for testing this class, can take an ARFF file as first argument.

    :   **Parameters:**: `args` - commandline options **Throws:**: `java.lang.Exception` - if something goes wrong in testing


---


|  |  |  |
| --- | --- | --- |
| **Overview** \| **Package** \| **Class** \| **Tree** \| **Deprecated** \| **Index** \| **Help** \| **Weka's home** | |  |
| **PREV CLASS**   **NEXT CLASS** | **FRAMES**    **NO FRAMES**     **All Classes** |  |
| SUMMARY: NESTED \| FIELD \| CONSTR \| METHOD | DETAIL: FIELD \| CONSTR \| METHOD |  |


---
